# Antilisterial Activity of Plantaricin UG1 during Manufacture of Zabady and Kareesh Cheese: Two Arabian Dairy Products

**Published:** 2008-12

**Authors:** Abdulluah A. Altalhi

**Affiliations:** *Biology Department, Faculty of Science, Taif University, Box. 888, Taif, Saudi Arabia*

**Keywords:** *Listeria monocytogenes* LMG 10470 (*L. monocytogenes*), *Lactobacillus plantarum* UG1 (*Lact. Plantarum*), plantaricin UG1, bacteriocin, Zabady, Kareesh Cheese

## Abstract

Here, plantaricin UG1 was shown to inhibit *Listeria monocytogenes LMG10470 (L. monocytogenes)* cells. The inhibition was not caused by lactic acid produced by the bacteriocin producing strain *Lactobacillus plantarum* UG1 (*Lact. plantarum*). Partially purified plantaricin UG1 had a higher antilisterial activity than *Lact. plantarum* cells in both Zabady and Kareesh cheese samples during their maturation. Compared to the antilisterial activity in Kareesh cheese, plantaricin UG1 showed a faster effect during Zabady manufacture.

## INTRODUCTION

*Listeria monocytogens* *(L. monocytogenes)* is a gram positive rod that is catalase positive and shows a characteristic tumbling mobility (1). It has long been recognized as a veterinary pathogen and in humans causes a disease known as listeriosis particularly in neonates or immunocompromised hosts (2, 3). A number of listerial outbreaks related to dairy products have been described (4). Two sporadic cases of listerial meningitis were also attributed to consumption of dariy products (5). *L. monocytogene*s is able to grow in dairy products at various acidic conditions (PH3.5-7) and at various incubations temperatures (10°C-45°C) (6). Hence, inhibition of *L. monocytogenes* by a safe lactic acid bacterium or its metabolites is of interest. In this regard the bacteriocin planetaricin UG1 produced by *Lact. pantarum* inhibited some food–borne pathogens including *L. monocytogenes in vitro* (3, 6, 7, 8, 9). The bacteriocin plantaricin UG1 was active against *L. monocytogenes* at acidic and neutral PH values (pH3.5-7) and over a wide temperature range (Zero-90°C) (10, 11, 12). The present work was undertaken to inhibit growth of *L. monocytogenes* during maturation of two Arabian dairy products (Zabady and kareesh cheese) by the bacteriocin plantaricin UG1.

## MATERIALS AND METHODS

**Cultures and media:** *L. monocytogenes* LMG10470 was provided from LMG culture collection, Laboratorium voor Microbiologie, Universiteit Gent, Belgium. It was counted on trypticase soy broth (13). *Lact. plantarum* UG1, the producer of plantaricin UG1 was provided kindly from prof. Dr. Gamal Enan at Botany Department, Faculty of Science, Zagazig University, Egypt. The pantaricin UG1 megative variant (*BAC*) was obtained as described previously (3, 8). *Lact. plantarum* UG1 and its *BAC* were grown in De Man, Rogosa and Sharpe medium (MRS) (14). Competitive growth of *L. monocytogenes* and *Lact. plantarum* UG1 cells (wild strain and *BAC* variant) were tested in BHI broth (Oxoid). Competitive growth of the zabady and Kareesh cheese starter cultures were also tested in BHI broth. Growth values were tested on agar plates (tryplic soy agar for listerias cells and MRS agar for *Lact. plantarum* and its *BAC* strain). The results were obtained from three replicates.

**Preparation of partially purified plantaricin UG1:** *Lact plantarum* was grown in MRS broth for 16 h at 30°C. Cell-free supernatant was abtained by centrifuging the culture (10000 xg. for 10 min at 4°C). The pH value of cell free supernatant was then adjusted to 6.5. This was to exclude the inhibitory activity due to organic acids. This pH-adjusted cell free supernatants were subjected to ammonium sulphate precipitation (50% saturation) as described previously (7). The ammonium sulphate precipitates (surface pellicels and pellets) were recovered in 10 mM potassium phosphate buffer, pH6.5, and dialyzed against the same buffer for 24 h at 4°C (13). The partially purified plantaricin UG1 was sterilized by filtration through cellulose membrane filters (0.45 µm, Milipore, Amicon). The activity of partially purified plantaricin UG1 was measured as descurbed previously. One of this partially purified plantaricia UG1 appeared to contain 22880 AU/ml.

### Inhibition of L. monocytoenes by bacteriocin producing Lact. plantarum and partially purified plantaricin UG1 during Zabady maturation

Zabady is an Arabian Yogurt. It was manufactured as follows: Mixture of fresh buffalo’s and cow’s milk (1:1) was heated at 80-82°C for 20 min. and cooled to 40°C. The milk was inculcated with 2% v/v of Zabady starter culture (mixture of *Lactonacillus bulgaricus* and *Streptococcus cremories*, Mircen Culture Collection, Ain Shams University, Egypt), mixed well, distributed to 150 ml scrow capped bottles. The bottles were then inoculated with about 2 × 10^6^ cfu/ml of *L. monocytogenes* and were classified into three series. First; second series were inoculated with about 2 × 10^6^ cfu/ml of *Lact. plantarum* (wild strain); *BAC* respectively. Third series of bottles were treated with about 22880 AU/ml of partially purified plantraricin UG1 (6). The contents of bottles were mixed well by hand Bottles were then incubated at 37°C for three days until curdled well. 5gm portions were taken at regular time intervals and analysed for viable cunts (cfu/ml) of *L. monocytogenes* as described above.

### Inhibition of L. monocytoenes by bacteriocin producing Lact. plantarum and partially purified plantaricin UG1 during during kareesh cheese manufacture

Kareesh cheese is an Arabian dairy product. It was made as follows: Fresh defatted cow’s milk was heated at 85°C for 10 min. and then cooled to room temperature (approximately 25°C). The sterile milk was inculcated with 5% v/v mixed starter culture of *Lactobacillus bulgaricus* and *Streptococcus cremories*, Mircen Culture Collection, Ain Shams University, Egypt. It was also inoculated with about 2 × 10^6^ cfu/ml of *L. monocytogenes*, mixed well and distributed to 2 L scrow capped plastic containers. Those 2 L samples were inoculated with about 2 × 10^6^ cfu/ml of either *Lact. plantaru*m (the wild strain) or *BAC*. Other series of 2 L samples were treated with about 22880 AU/ml of partially purified plantraricin UG1 (8). Samples were incubated at 37°C until coagulation (2 days). The curd was scooped into mould (s) and was left to drain for 8 days. 5 g portions were removed every day throughout 10 days of maturation (before and after curd formation). Serial dilutions were prepared and viable counts (cfu/ml) of *L. monocytogenes* were determined.

## RESULTS

In a preliminary experiment, growth of *L. monocytogenes* in BHI broth inoculated with either Zabady starter or Kareesh cheese starter cultures were tested. The results showed nearly comparable growth values of listerias cells in the absence of starter cultures. Growth of *L. monocytogenes* in Zabady samples (during maturation) inoculated by either *Lact. plantarum* or *BAC* strain were tested. Results are given in Table 1. *L. monocytogenes* alone (control) was increased from 2 × 10^6^ cfu/ml to 9.7 × 10^9^ cfu/ml within 4 days, whereas listerias growth was inhibited in presence of *Lact. plantarum* (the bacteriocin producer strain) reaching 7.7 × 10^3^ after 72 h and 1.1 × 10^2^ after 96 h. The *BAC* strain failed to inhibit *L. monocytogenes* and growth values of listerias cells were comparable to *Lact. Plantarum*. Addition of partially purified plantaricin UG1 to the raw milk at the beginning of Zabady manufacture resulted in a marked bactericidal effect with reduction in viable counts of *L. monocytogenes* by four log cycles after 72 h (Table 2). No growth of lysterias cells was obtained after 84 h (Table 2). However, the difference in colony counts between the treated samples and controls increased to reach 9 × 10^9^ cfu/ml after 84h (Table 2).

**Table 1:** Growth of *L. monocytogenes* during maturation of an Arabian Zabady samples inoculated by the bacteriocin producer strain: *Lact. plantarum*

Time (h)	Control without *Lact. plantarum.* UG1	In the presence of	
		*Lact. plantarum*	*BAC*

6	8 × 10^6^	6.2 × 10^6^	7.6 × 10^6^
12	2 × 10^7^	1.1 × 10^6^	1.1 × 10^7^
24	3.1 × 10^8^	1 × 10^6^	8.8 × 10^7^
36	8.7 × 10^8^	2.6 × 10^5^	2.1 × 10^8^
48	1.1 × 10^9^	1.1 × 10^5^	9.2 × 10^8^
60	5.8 × 10^9^	8 × 10^4^	1 × 10^9^
72	6.8 × 10^9^	7.7 × 10^3^	3.1 × 10^9^
84	9 × 10^9^	3 × 10^3^	5 × 10^9^
96	9.7 × 10^9^	1.1 × 10^2^	8.1 × 10^9^

**Table 2:** Growth of *L. monocytogenes* during maturation of an Arabian Zabady samples treated with the bacteriocin plantarcin UG1

Time (h)	Zabady samples without plantarcin UG1 (control)	Zabady samples inoculated with plantaricin UG1

6	8 × 10^6^	1.1 × 10^5^
12	2 × 10^7^	8.6 × 10^4^
24	3.1 × 10^8^	3.3 × 10^4^
36	8.6 × 10^8^	3.1 × 10^4^
48	2 × 10^9^	3 × 10^3^
60	5.7 × 10^9^	2.1 × 10^2^
72	6.8 × 10^9^	1.1 × 10^2^
84	9 × 10^9^	Zero
96	9.3 × 10^9^	Zero

As given in Table 3, inoculation of the raw milk used for Kareesh cheese at the beginning of manufacture processes resulted in a gradual decrease of the viable cell population of *L. monocytogenes* reaching 2.3 × 10^4^ cfu/ml after 4 days and 9.3 × 10^3^ cfu/ml after 6 days and 2 × 10^2^ cfu/ml after 10 days whereas growth of *L. monocytogenes* alone (control) was increased from 2 × 10^6^ cfu/ml to 2 × 10^9^ cfu/ml within 10 days. A significant bactericidal effect of partially purified plantaricin UG1 was obtained during maturation of Karesh chesse samples (Table 4). No growth of listerias cells was observed in Kareesh cheese after 7 days of storage. However, growth of *L. monocytogenes* cells in controls was increased from 2 × 10^6^ cfu/ml to 2 × 10^9^ cfu/ml within 10 days.

**Table 3:** Growth of *L. monocytogenes* during maturation of samples of an Arabian Kareesh cheese inoculated with the bacteriocin producer strain *Lact. Plantarum* UG1

Time (day)	Samples of Kareesh cheese without *Lact. plantarum* (control)	Samples of Kareesh cheese Inoculated by	
		*Lact. plantarum* UG1	*BAC*

zero	2 × 10^6^	2 × 10^6^	2 × 10^6^
1	7 × 10^7^	3.2 × 10^5^	7 × 10^7^
2	3 × 10^8^	1.1 × 10^5^	2.9 × 10^8^
3	1.1 × 10^9^	6.6 × 10^4^	1 × 10^9^
4	1.3 × 10^9^	2.3 × 10^4^	1.3 × 10^9^
5	1.1 × 10^9^	1.1 × 10^4^	1.1 × 10^9^
6	2.2 × 10^9^	9.3 × 10^3^	2.2 × 10^9^
7	3.3 × 10^9^	6 × 10^3^	1 × 10^9^
8	2.2 × 10^9^	6.2 × 10^3^	9.9 × 10^8^
9	1.2 × 10^9^	3.3 × 10^3^	8.6 × 10^8^
10	2 × 10^9^	2 × 10^2^	8 × 10^8^

**Table 4:** Growth of *L. monocytogenes* during maturation of an Arabian Kareesh cheese samples treated with plantarcin UG1

Time (day)	Kareesh Cheese samples without plantarcin UG1 (control)	Kareesh Cheese samples treated with plantaricin UG1

Zero	2 × 10^6^	2 × 10^6^
1	6.7 × 10^7^	5 × 10^5^
2	3.1 × 10^8^	4 × 10^4^
3	1.2 × 10^9^	1.3 × 10^3^
4	3 × 10^9^	2 × 10^2^
5	1.2 × 10^9^	1 × 10^2^
6	2.1 × 10^9^	1 × 10^2^
7	1.6 × 10^9^	Zero
8	2.2 × 10^9^	Zero
9	2.1 × 10^9^	Zero
10	2 × 10^9^	Zero

## DISCUSSION

Bacteriocins are a group of antimicrobial proteins produced by Gram-positive bacteria (15). Use of lactic acid bacteria bacteriocins has been suggested a way to prevent spoilage in dairy products (16). The only bacteriocin produced by lactic acid bacteria and currently used in dairy industry is nisin which is produced by *Lactococcus Lactis* ssp *lactis* and has a limited applicability because of its instability at neutral pH (17). In an attempt to fill this gap, our results showed that plantaricin UG1 produced by *Lact. plantarum* has a vigorous antiliterial activity In Zabady and Karesh cheese samples during their maturation.

The experiments employed herein were designed to concur with the conditions that occasionally occur during maturation of either Zabady or Kareesh cheese which is often attained at room temperature in the Arabian countryside. The plantarcin UG1 concentration (22880 AU/ml) used in this study was choosed because it was appeared to be an ideal inhibitory one against *L. monocytogenes* (3).

The inhibition of listerias cells observed in this study was due to plantaricin UG1, and not to lactic acid produced by *Lact. plantarum* as the growth values of listerias were unaffected by the plantaricin UG1 negative variant (*BAC*). This is consistent with many previous investigations (3, 8, 15, 18, 19). Partially purified plantarcin UG1 had a higher antilisterial activity than *Lact. plantarum* UG1 cells in both Zabady and Kareesh cheese at maturation. This could be due to adsorption of plantaricin UG1 to both live and dead cells of its producer strain: *Lact. Plantarum* (3, 8, 9, 20).

Compared to the antilisterial activity in Kareesh cheese, plantaricin UG1 showed a faster effect in Zabady samples during their maturation. This may be due to increased diffusion of this bacteriocin in Zabady as compared to the more solid curd of Kareesh cheese. These results are in agreement with previous published results (13).

The antilisterial activity of plantaricin UG1 observed in this study during either Zabady or Kareesh cheese manufacture is quite promising for development of a wider application of the bacteriocin plantaricin UG1 in the dairy industry. In addition, plantaricin UG1 did not affect growth of starter lactic acid bacteria in either Zabady or Kareesh cheese during their maturation and hence this bacteriocin (plantaricin UG1) will be an ideal dairy preservative. This also could replace or reduce the use of dangerous chemical additives.
